# Mobile Robot Self-Localization with 2D Push-Broom LIDAR in a 2D Map

**DOI:** 10.3390/s20092500

**Published:** 2020-04-28

**Authors:** Jordi Palacín, David Martínez, Elena Rubies, Eduard Clotet

**Affiliations:** Robotics Laboratory, University of Lleida, Jaume II, 69, 25001 Lleida, Spain

**Keywords:** 2D push-broom LIDAR, tilted-down 2D LIDAR, mobile robot self-location, 2D map

## Abstract

This paper proposes mobile robot self-localization based on an onboard 2D push-broom (or tilted-down) LIDAR using a reference 2D map previously obtained with a 2D horizontal LIDAR. The hypothesis of this paper is that a 2D reference map created with a 2D horizontal LIDAR mounted on a mobile robot or in another mobile device can be used by another mobile robot to locate its location using the same 2D LIDAR tilted-down. The motivation to tilt-down a 2D LIDAR is the direct detection of holes or small objects placed on the ground that remain undetected for a fixed horizontal 2D LIDAR. The experimental evaluation of this hypothesis has demonstrated that self-localization with a 2D push-broom LIDAR is possible by detecting and deleting the ground and ceiling points from the scan data, and projecting the remaining scan points in the horizontal plane of the 2D reference map before applying a 2D self-location algorithm. Therefore, an onboard 2D push-broom LIDAR offers self-location and accurate ground supervision without requiring an additional motorized device to change the tilt of the LIDAR in order to get these two combined characteristics in a mobile robot.

## 1. Introduction

The fundamental tasks for autonomous mobile robots are collision avoidance and self-localization mainly based on the use of onboard ultrasonic sensor systems, vision-based systems or light detection and ranging (LIDAR) sensor systems. One method widely used to implement the self-location task is simultaneous localization and mapping (SLAM) [1] which usually combines the information of different onboard sensors [2] in order to create a map of the environment and to track the trajectory of the mobile robot. The SLAM method covers a wide list of subtasks and alternatives and allows the development of fine algorithm enhancements [3].

In general, the use of a fixed horizontal 2D LIDAR in a mobile robot [4] produces a point cloud representation of a horizontal 2D plane of the environment. This point cloud does not include objects that do not have a trace in the measurement plane, such as the hole of a stairway going down or the shape of a small object placed in the ground. This problem can be addressed using a motorization to tilt down/up, spin/roll or nod/pitch a 2D LIDAR, or combining several 2D LIDARs, or using specific 3D LIDARs. Any of these solutions will enhance the implementation of collision detectors while also enabling the 3D reconstruction of the environment [5]. However, the inclusion of these solutions in a mobile robot increases its control complexity and cost which is a competitive factor that precludes the commercial development of task such as table cleaning [6] or teaching assistance [7].

There are a large number of studies based on developing and using a motorized 2D LIDAR. For example: Lit et al. [8] proposed a system to obtain 2D scans at various angles reaching the conclusion that a calibration method is needed in order to extend a single-layer 2D LIDAR to a multi-layer LIDAR because the tilt axis is not passing through the center of all the measurement planes. Ohno et al. [9] proposed a pan-tilt system to develop a 3D scanner while detecting moving objects concluding that those mechanisms need a calibration in order to obtain reliable scans during the tilt or pan movement. Kang et al. [10] proposed a calibration method to estimate the six degree-of-freedom of a rotating 2D LIDAR platform. Zeng et al. [11] proposed an improved calibration method for a rotating 2D LIDAR platform in order to resolve the bias originated by the mechanical displacement of the LIDAR. Alismail et al. [12] proposed a fully automated algorithm in order to calibrate a spinning/rolling 2D LIDAR. Yamao et al. [13] proposed a calibration method in order to obtain 3D scans based on identifying the same object on 2D scans. The conclusion is that a calibration is needed when combining multiple sensors [14]. Finally, Morales et al. [15] proposed a 3D laser scanner based on pitching a 2D LIDAR while maintaining the optical center of the different 2D measurement planes, solution adopted also in this paper.

The proposal of this paper is inspired on the work of Takahashi et al. [16] which proposed the development of an emergency obstacle avoidance for a mobile robot by using a fixed push-broom 2D LIDAR sensor. The term push-broom describes the scanning strategy of the sensor, by which it is tilted to the ground, as opposed to its common horizontal usage. This push-broom application has the advantage of detecting and avoiding low-height obstacles and slopes in the trajectory of the mobile robot that would not be detected by a fixed horizontal 2D LIDAR. Inspired on this work, the new contribution of this paper is the evaluation of the hypothesis that a 2D push broom LIDAR can be also used to self-locate the position of a mobile robot using as a reference a previously obtained horizontal 2D map created with SLAM procedures and the same 2D LIDAR. This hypothesis is based on the assumption that a conventional facility usually has fixed walls, doors and different big furniture objects that are not prone to changes. Therefore, it is supposed that a reference 2D map of the environment obtained at a fixed height will be valid for a long period of time, reducing the need of creating new maps or updating already existing ones for the 2D mobile robot self-location procedure. This new proposal can be used to include SLAM in simple mobile robots using a fixed 2D push-broom LIDAR for obstacle avoidance, in transversal precision agriculture applications where a fixed 2D LIDAR is mainly used to gather information about plant architecture [17,18,19], and in combining SLAM and obstacle avoidance in mobile robots carrying a motorized 2D LIDAR.

The paper is structured as follows: Section 2 presents the materials and methods. Section 3 presents a set of experiments designed to verify the detection performances and to obtain an optimum 2D LIDAR tilt angle. Section 4 presents the results of self-localization experiments. Finally, Section 5 outlines the discussion and conclusions of the paper. The raw 2D scans used in the experimental part of this paper are available to download as supplementary MATLAB data files (.mat) which can be visualized through a menu-driven MATLAB function (main.m) that allows an easy selection of the different cases and a direct visualization of the scanned point clouds.

## 2. Materials and Methods

The main materials and methods used in this paper are a LIDAR mounted on the mobile robot assistant personal robot (APR), a 2D reference map already available, and the iterative closest point (ICP) self-location algorithm.

### 2.1. Mobile Robot APR

The mobile robot used to experimentally validate the main hypothesis of this paper is a human-size assistant personal robot (APR) created as a family of versatile research tools for laboratory experimentation. The concept of the APR was firstly presented by Clotet et al. [20] and was based on a three-wheel holonomic motion system [21] with a height of 1680 mm, a diameter of 540 mm and a total weight of 38.3 kg. The initial design of this mobile robot was improved with a suspension system in order to reduce the transmission of vibrations to the head of the mobile robot [22]. Several prototypes of the APR family have been used in different applications. Clotet et al. [20] proposed the use of an APR robot as a tele-operated assisted living tool. Martinez et al. [23] used an APR to develop an ambient intelligence application. Palacin et al. [24] developed an automatic measurement system to supervise temperature, humidity and luminance with an APR. Palacin et al. [25] proposed the development of a MOX sensor system for the APR mobile robots to detect gas leaks in indoor environments. Finally, Palacin et al. [26] proposed the application of the APR as a walker-helper tool, taking advance of the size and weight of the mobile robot in order to operate as a physical support for users with reduced mobility during indoor displacements.

**Figure 1:**
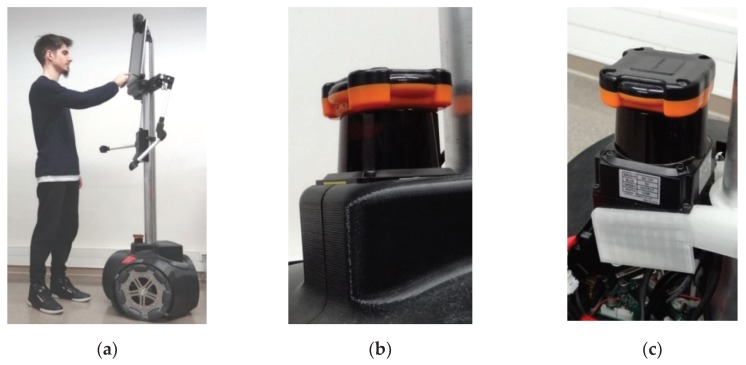
(**a**) APR-03 mobile robot operated by an author of this paper. (**b**) Close-up image of the onboard LIDAR sensor. (**c**) Detail of the onboard LIDAR without the protective housing cover.

Figure 1a shows an image of the APR model 03 (APR-03) interacting with a researcher of this paper. The mobile robot has a tactile screen which is used for human-robot interaction and also for visual human communication through a minimalistic, line-based animated face. The mobile robot has two articulated arms with rigid aesthetic hands, mainly used as a non-verbal communication reinforcement tool. Figure 1b shows a close-up image of the onboard 2D LIDAR in the APR-03 and Figure 1c shows the 2D LIDAR without the plastic protective cover.

### 2.2. 2D LIDAR: UTM-30LX Model

Figure 1b shows the Hokuyo UTM-30LX, a 2D LIDAR sensor manufactured by Hokuyo (Osaka, Japan) mounted in the mobile robot APR-03. The UTM-30-LX model is a compact (60 × 60 × 87 mm) and lightweight (210 g) LIDAR device widely used in robotic applications [27]. This indoor 2D LIDAR is composed of a fixed 905 nm semiconductor laser diode and a stepper motor to rotate a reflecting mirror that rotates the laser beam around the device which operates as a Class 1 laser device (safe under normal conditions of use). The UTM-30LX device has a distance measurement range from 100 mm to 30,000 mm with an accuracy between ±30 mm and ±50 (depending on lighting conditions), and an angular field of view of 270° with an angular resolution of 0.25°. The UTM-30LX requires 25 ms to perform a complete rotation of the inner laser beam, generating a sequence of up to 1081 distance points per scan when covering the complete field of view (270°).

### 2.3. 2D Reference MAP

APR mobile robots use a UTM-30LX LIDAR for SLAM and trajectory tracking. These mobile robots can be configured either (1) to apply SLAM from scratch in order to create a new 2D map of a new exploration area, (2) to apply SLAM using as a reference a previously generated 2D map (this procedure allows maps to be updated to include new obstacles and the interior of rooms that were not accessible during the first mapping process) and (3) to apply SLAM using a previously generated 2D reference map (that can be shared between the mobile robots) without updating this 2D map at the end of the SLAM procedure. The only difference between all these procedures is the final step of updating the current 2D map with new information provided by the 2D LIDAR. In all cases, the SLAM procedure is based on the iterative closest point (ICP) algorithm [28,29].

**Figure 2:**
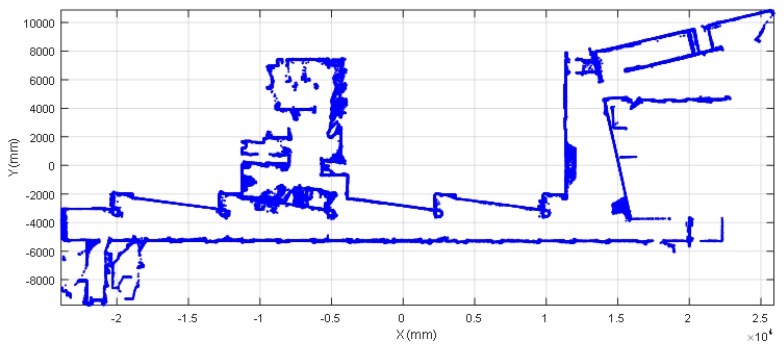
Reference point cloud map of the experimentation facility.

The 2D map used in this paper was created by the authors in [25] and represents the second floor of the Escola Politècnica Superior of the University of Lleida. This large experimentation facility includes a combination of different elements that can represent a challenge for self-location procedures that rely on the use of the data gathered by a laser-based 2D LIDAR sensor (high-reflective surfaces, translucent objects, small obstacles…). Some of the conflictive elements that are present in the designed area are: transparent glass walls, semi-translucent windows, thin handrails, benches with thin legs, etc. Figure 2 shows a point cloud representation of the reference 2D map used in this paper (also included in the supplementary files of the paper). This original map was created specifically to include the area of one laboratory and two offices of the floor as regions of interest or regions of operation for the application of the APR-03 described in Palacin et al. [25].

### 2.4. ICP Self-Location Algorithm

The ICP [28] is an algorithm mostly used in mobile robots to virtually reconstruct 2D and 3D scenarios [29] and track its position and orientation in the scenario. The mobile robot APR-03 [25] performs SLAM using the ICP algorithm to process the 2D scanning point clouds gathered from an onboard UTM-30LX 2D LIDAR placed horizontally in front of the mobile robot (Figure 1) at a relative height of 380 mm. The result of the SLAM procedure was the creation of a horizontal 2D map (a horizontal slice of the facility) (Figure 2) and the tracking of the displacement of the mobile robot in this 2D map.

The ICP algorithm has to deal with the problem of having a set of individual scanning point clouds that contain shared information of the scenario without a common coordinate system. This uncertainty is caused due to the fact that each point cloud is unaware of the position of the sensor in the real world during a scanning process, assuming that each scan is taken at the origin of the coordinate system. In general, the ICP algorithm tries to establish the position/orientation difference between an initial scanning point cloud or map and the current scanning point cloud by continuously computing a rotation/transformation matrix that minimizes the distance between the point clouds until the distance between them is lower than a specific threshold, or until a maximum number of iterations is reached. This computation allows the definition of a common coordinate system and origin between all the point clouds in order to describe the scenario and the current position of the mobile robot in it.

The ICP algorithm takes advantage of the presence of redundant information between consecutive point clouds in order to calculate the position and orientation difference between them. This redundancy is given by the assumption that two consecutive scanning point clouds will contain common region information of the scenario but observed from a different perspective (position/orientation). The ICP algorithm includes the following internal steps: point selection, neighborhood selection, point matching, weighting, rejection and minimization that can be performed using a wide variety of strategies. Donso et al. [30] presented a study showing the results obtained when using different strategies for each one of the steps of the ICP algorithm while mapping the layout of a mining operation. Regarding the objective of this paper, the minimization step of the ICP algorithm can be implemented using two main strategies depending on the metric used to determine the convergence error between the 2D map and the current point cloud: point to point and point to plane.

The ICP algorithm implemented in the SLAM procedure defined for the APR mobile robots use the point to plane strategy. In general, it is well known that point to plane is a noticeably slower metric than point to point due to its complexity [31]. However, in an indoor scenario, point to plane provides better results when merging scanned point clouds that are mostly composed of straight lines (such as the large walls that conform the explored area) because it tries to align the current point cloud with the nearest planes of the 2D map rather than trying to reduce the distance between the point clouds to zero like the point to point.

## 3. 2D Push-Broom LIDAR: Verification of the Detection Performances

The main hypothesis of this paper is that the data gathered with a 2D push-broom LIDAR can be used in a mobile robot for self-location using a 2D reference map previously created with a 2D horizontal LIDAR. 

This configuration must detect also low-height objects, ramps and holes usually not detected by a horizontal LIDAR [16] which only provides information on what is present at a certain height (Table 1). Then, the questions related with a 2D push-broom LIDAR application are: (1) the verification of the detection capabilities, and (2) the determination of the optimum tilt angle.

A set of detection experiments have been proposed in order to verify the detection capabilities of a 2D push-broom LIDAR and to estimate an optimal tilt angle. In all these experiments some objects have been placed at multiple distances in front of the mobile robot which remains static. The height of the LIDAR was fixed to 380 mm and its tilt angle was manually changed from 0° (horizontal) to 45° (limited by the structure of the mobile robot) in increments of 5°. Table 1 shows different illustrative images obtained during the development of the verification experiments. These objects are: a small box (225 × 80 × 155 mm), one shoe/leg, stairs going down and stairs going up. Based on our experience, these objects are relevant obstacles that must be detected by a mobile robot.

**Figure 3:**
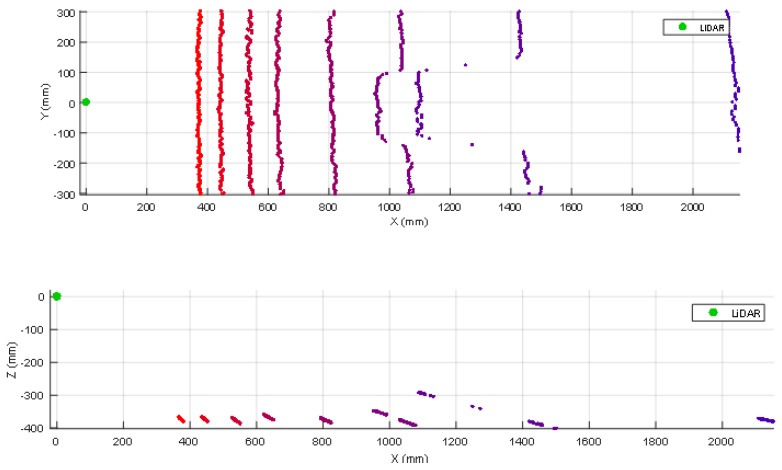
Top (up) and lateral (down) view of different scans obtained when placing a small box on the ground at approximately 1000 mm from the LIDAR. The height of the LIDAR was 380 mm and the tilt was changed from 45° (reddish ground line, close to X = 0) to 80° (violet ground line) in increments of 5°.

The use of a tilted-down 2D LIDAR provides information of the ground plane in front of the mobile robot. In all the experiments the ground appears as a frontal line that has some measurement noise (see Figure 3). Then, an obstacle placed in the ground produces closer distance points that breaks the continuity of the ground line (see Figure 3). The automatic detection of an obstacle has been carried out by applying a ground distance threshold of ±50 mm after rotating and translating the scan according to the angle and height of the LIDAR sensor, causing the ground points to have a height of approximately 0 mm. This threshold value was tuned by trial and error. For example, if after applying to the scan the previously mentioned transformation (rotation/translation) the Z value of a point is lower than −50 mm the point will be classified as a hole, if its Z value is greater than 50 mm it will be classified as an obstacle; otherwise, the point will be classified as ground. This procedure does not provide information of the type of the object: foot, leg, box, furniture, etc.

Other obstacle cases fully considered and analyzed but not included on this paper are a medium size box, detected similarly to a small box; a big box, detected as an obstacle; a small paper bin, which is not detected by the 2D LIDAR in its original configuration (only detected when the tilt is 5° or higher); other shoe/leg cases with different textures and colors, detected similarly as the one shoe/leg case; and a static person in front of the robot (appearing in the scans as two parallel shoes or two legs), detected similarly as the one shoe case.

### 3.1. Detection of a Small Box

The case of a small box (or other kind of small object) placed on the ground in front of a mobile robot will never be detected by a horizontal 2D LIDAR with a measurement plane higher than the height of the object. Figure 3 summarizes two 3D views of some scans obtained when placing a small box in the ground in front of the mobile robot at approximately 1000 mm from the LIDAR.

In this experiment the 2D LIDAR has been tilted from 0° (horizontal) to 45° (limited by the structure of the mobile robot) in increments of 5° but, for simplicity, Figure 3 shows only the range from 0° to 80°. Figure 3 shows the ground lines and broken ground lines when the scan covers the object. Finally, Table 2 summarizes the complete list of LIDAR distances and tilt angles experimentally evaluated. In general, the small box is correctly detected in a distance range from 4000 mm to 250 mm with a tilt angle from 5° to 45°. Table 2, label (√) means that the object has been correctly detected. Table 2, label (R) means that the frontal part of the box has been detected after a reflection of the laser beam with the ground so the object can be misclassified as a hole in the ground because the effective distance measured by the LIDAR is higher and under the ground level due to the effect of the reflection.

### 3.2. Detection of One Shoe/Leg

**Figure 4:**
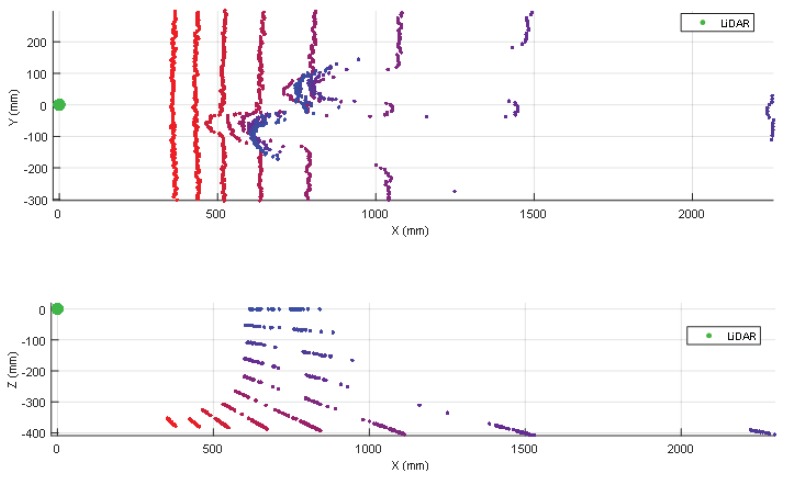
Top (up) and lateral (down) view of the different scans obtained when placing one shoe/leg at a fixed distance from the LIDAR. The tilt of the LIDAR has been changed from 0° (horizontal) to 45° in increments of 5°.

The case of one shoe/leg (from a moving or unaligned person) is apparently not a challenging detection because a person is normally tall enough to cross the line of sight of a 2D LIDAR mounted on a mobile robot regardless of its position and orientation. However, the one shoe/leg case analyzed (see Table 2) includes the use of black and soft clothes that reduces laser reflectivity and increases distance variability. Figure 4 shows two 3D views of the 2D scans corresponding to one person with a shoe/leg located at 500 mm from the LIDAR (the second shoe/leg is behind). Figure 4 shows that a shoe/leg breaks the expected line of the ground, so it is detected as an object in front of the mobile robot. Table 3 summarizes the complete list of LIDAR distances and tilt angles evaluated; in general, one shoe/leg is correctly detected in all distance range and all tilt angles considered. The information gathered by the 2D LIDAR is used to avoid collisions with static persons. Additionally, the initial design of the APR also considered the possibility of a user colliding with the mobile robot. For this, the robot includes a soft plastic cover that can absorb part of the impact, preventing direct contact between the person and the inner metallic structure of the robot in case of collision.

### 3.3. Detection of Stairs Going Down

**Figure 5:**
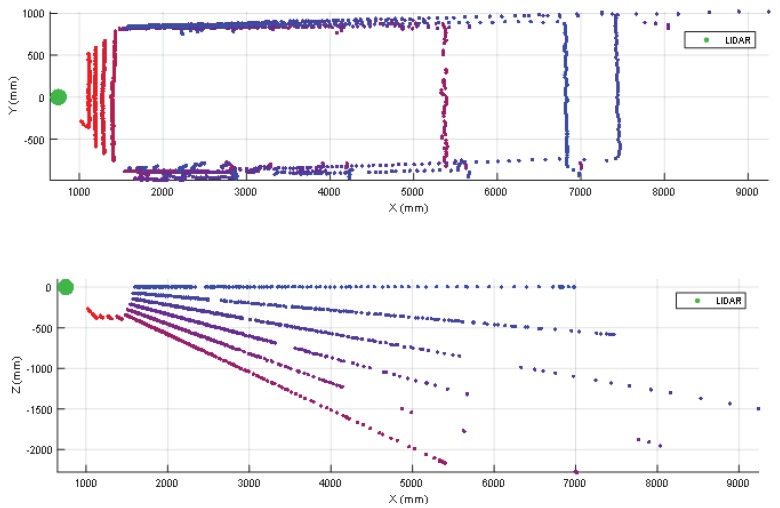
Top (up) and lateral (down) view of the different scans obtained when having stairs going down at a fixed distance of 1250 mm depending on the 2D LIDAR tilt angle. The tilt of the LIDAR has been changed from 0° (horizontal) to 45° in increments of 5°. The distance points originated by the surrounding walls difficult the visual interpretation of the data shown.

The case of stairs going down will never be detected by a horizontal 2D LIDAR. This fact represents a challenge for a fast and heavy mobile robot such as the APR-03 because a floor sensor located under the structure of the mobile robot will not be able to warn and stop the mobile robot in time before falling down the stairs due to its inertia. Figure 5 shows two 3D views of the scans corresponding to a stair going down located at 1250 mm from the LIDAR. In this case there are no scan points at the position where the line of the ground should be. Then, Table 4 summarizes the complete list of distances and tilt angles experimentally evaluated. In this case the numeric value represents that an object is detected at the labeled distance, the label (S) means that a big hole is detected probably because of a stair going down, and the label (G) means that the ground has been detected at its expected location. In this experiment, a tilt of 0° provides the detection of a far frontal object (which is the shape of the complementary stair going up) so the large hole of the stairs will remain wrongly and dangerously undetected by the mobile robot. Alternatively, a tilt of 35° or higher reduces the effective distance at which the hole of the stairs can be detected by the robot before falling down. In any case, the results of this experiments show that an unknown stair going down (not registered as a dangerous area in a reference 2D map) represents a challenging situation for a mobile robot using only a horizontal 2D LIDAR for self-location and trajectory planning.

### 3.4. Detection of Stairs Going Up

**Figure 6:**
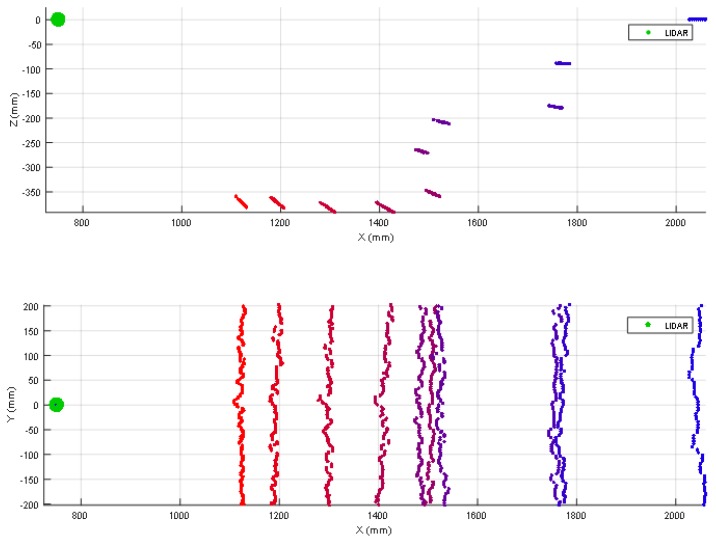
Top (up) and lateral (down) view of the different scans obtained when having stairs going up at a fixed distance of 750 mm in front of the 2D LIDAR depending on tilt angle. The tilt of the LIDAR has been changed from 0° (horizontal) to 45° in increments of 5°. The distance points originated by the surrounding walls have been eliminated for enhanced visual interpretation.

Figure 6 shows two 3D views of the 2D scans corresponding to a stair going up located at a relative distance of 750 mm from the LIDAR. The scan points depicting the walls have been removed from this representation in order to enhance the visual interpretation of the data. In this example case, the obstacle that represents the stairs is detected with LIDAR tilt angles from 0° (horizontal plane) to 35°. However, when the tilt angle was 0° the relative distance to the object was higher than 1300 mm, and around 1000 mm for tilt angles from 5° to 10° so the distances detected with these tilt angles does not reveal the real obstacle that represents the stairs.

Table 5 summarizes the complete list of distances and tilt angles evaluated. In this case the numeric value represents the detection of an object at the labeled relative distance, the label (G) means that the ground has been properly detected at its expected location. The results of this experiments show that an unknown stair going up (not registered as a dangerous area in a reference 2D map) is usually detected at a distance farther than reality. Therefore, the stair going up represents always a challenging obstacle for a mobile robot using only a horizontal 2D LIDAR.

### 3.5. Optimal Fixed 2D LIDAR Tilt Angle

The detection experiment results shown on Table 2, Table 3, Table 4 and Table 5 permits a discussion of the optimal fixed tilt angle of the 2D LIDAR mounted on the mobile robot APR-03. This optimal tilt angle must guarantee the detection of all frontal obstacles in order to avoid any collision or falls.

**Figure 7:**
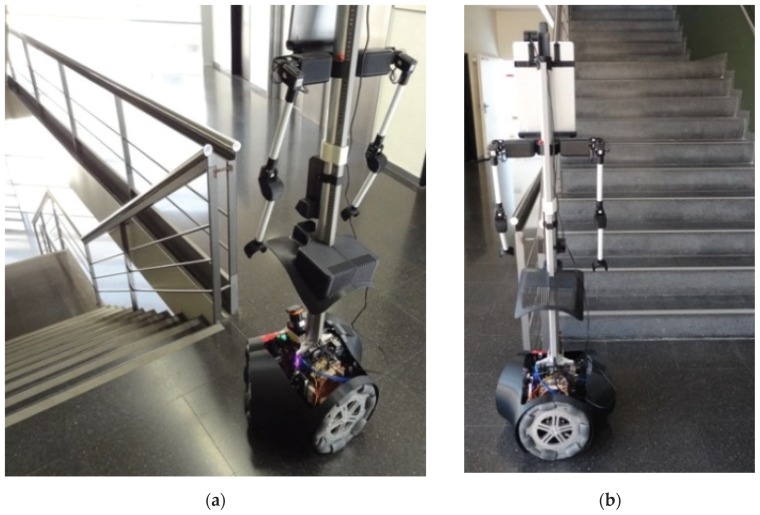
Mobile robot APR-03 placed in front of: (**a**) a stair going down and (**b**) a stair going up.

**Figure 8:**
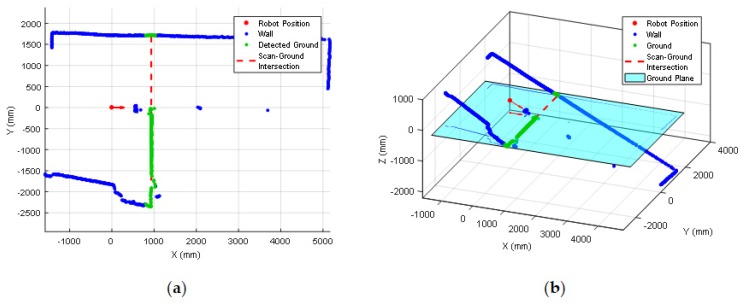
Experiment in front of a stairs going down: (**a**) raw scan data, (**b**) 3D representation. Distance points classified as ground (green), expected location of the ground (dotted red line).

**Figure 9:**
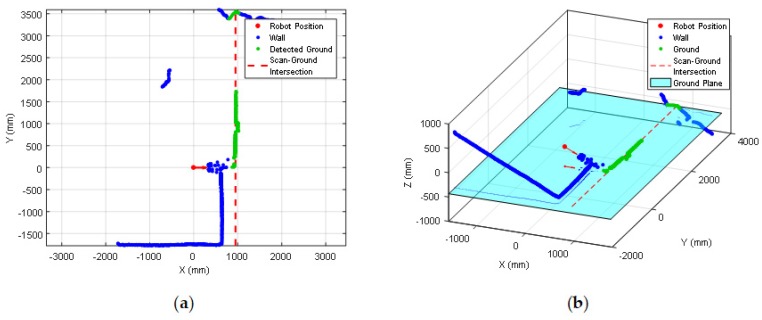
Experiment in front of a stairs going up: (**a**) raw scan data, (**b**) 3D representation.

The mobile robot APR-03 can go forward at a velocity up to 1 m/s. At the maximum velocity, the obstacles must be detected at around 450 mm in order to stop the mobile robot just before the collision. This minimum detection distance has been increased with an additional safety margin of 250 mm. Therefore, the 2D push-broom LIDAR must detect obstacles at a frontal distance of 700 mm from the mobile robot and the fixed tilt-down angle that guarantees this detection is 25°. The adequacy of this optimal fixed LIDAR tilt angle has been validated by performing pseudo-random repetitive displacements in the area of the stairs of the facility: going forward a random distance between 200 and 2000 mm (or until a frontal object is detected) plus a rotation with a random angle between −180° and +180°. The development of the validation experiments have been supervised by the members of our research group under controlled and safe conditions, ensuring the absence of people in the experimentation area and also having a remote control device to perform an emergency stop if needed. In all these experiments, the 2D reference map does not include any navigation restrictions or additional annotated information to prevent the robot from attempting to navigate across dangerous areas such as staircases or underneath chairs. In all validation experiments the detection of the stair going up and the stair going down has been successful and the proposed safety margin seemed reasonable and adequate. Figure 7 show two starting positions corresponding to two of this validation experiments in which the mobile robot APR-03 was placed in front of the stairs going down and up. Figure 8 shows a 2D and 3D view of the raw scan data points gathered by the UTM-30LX tilted down 25° when the mobile robot APR-03 was placed in front of the stairs going down: the origin of the coordinate system (0,0) and (0,0,0) is the location of the 2D LIDAR, the red arrow represents the location and orientation of the 2D LIDAR, the dotted red line represents the expected ground line, the green dots are the scan points detected as ground. In this case a big hole has been detected just in front of the mobile robot so this dangerous situation has been correctly detected and avoided by the mobile robot. Figure 9 shows also a 2D and 3D view of the raw scan data points gathered by the 2D LIDAR when the mobile robot was placed in front of the stairs going up. In this case the first step of the stairs has been detected as a near object in front of the mobile robot so the possible collision with the stair has been correctly detected and avoided.

These verification experiments have been the first experiments in which a mobile robot of the APR family has been configured to operate autonomously in the area of the stairs of the facility. In all of our previous experiments the APR mobile robots used the 2D LIDAR in horizontal for self-location in order to avoid this area. Therefore, this push-broom configuration can represent a huge improvement of the application capabilities of the APR mobile robot in a human compatible scenario in case of validating the utility of this push-broom LIDAR configuration also for self-location.

## 4. Self-Location Results

The hypothesis of this paper is that an already available 2D map created with a 2D horizontal LIDAR carried out by a mobile robot or by another mobile device in a specific exploration operation can be used to locate the position of a mobile robot using the same 2D LIDAR tilted down 25°, which is the optimal tilt angle obtained experimentally in the previous section of this paper for the mobile robot APR-03.

**Figure 10:**
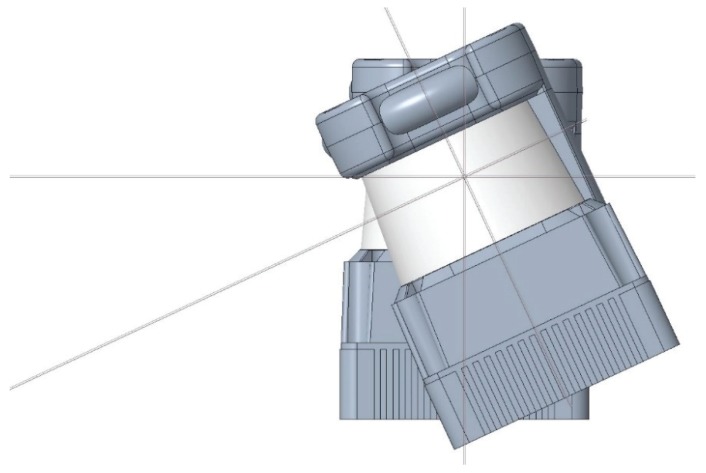
Illustration of the strategy applied to tilt down 25° the onboard 2D LIDAR. The horizontal line depicts the location of the measurement plane of the laser beam.

A specific set of experiments have been carried out to validate the self-location capabilities of the mobile robot APR-03 with the 2D LIDAR tilted down. In these experiments the mobile robot APR-03 will perform pseudo-random displacements and will use the 2D reference map created by the same mobile robot in a previous experiment (see Section 2.3). A special effort has been made to tilt down 25° the 2D LIDAR and maintain a common measurement axes with the reference horizontal (0°) orientation [15] (see Figure 10) in order to avoid any additional calibration [8].

**Figure 11:**
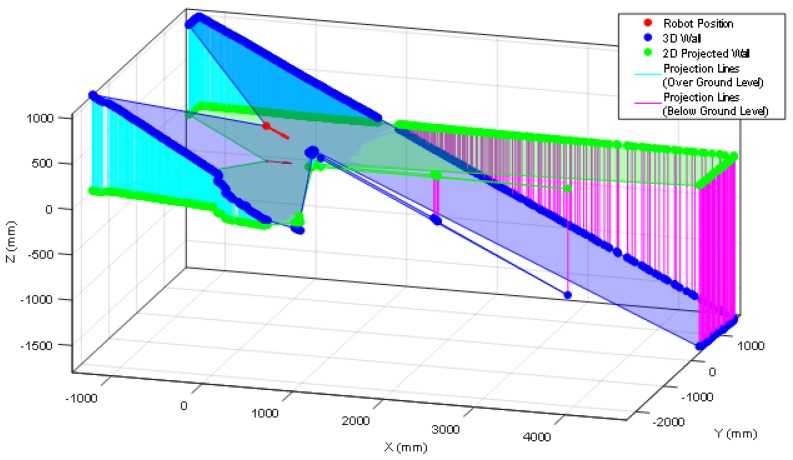
Original 2D scanned point cloud (blue) and its 2D projection in the horizontal plane of the reference map (green). The lines depicts the correspondence between the original and projected scan points: (light blue) cases over the ground level and (magenta) cases under the ground level.

The push-broom configuration requires pre-processing the 2D scan data gathered by the LIDAR before applying the ICP algorithm for self-location which consist on converting the 2D scan data in a 3D point cloud, removing the ground and ceiling points from the 3D point cloud (because the reference map does not include such information), and the projection of the 3D point cloud in the horizontal 2D plane used by the reference map in order to apply ICP. The complete pre-processing procedure has the following steps: (1) get one complete 2D scan data from the LIDAR, (2) delete the distance points lower than 20 mm as they are measurement artifacts, (3) use the height of the measurement plane of the LIDAR and the tilt angle to convert the 2D scan data in a 3D point cloud (see Figure 11), (4) delete the ground points in the 3D point cloud (points with a height between 50 and −50 mm), (5) delete the ceiling points in the 3D point cloud (points with a height higher than 2430 mm), (6) project the remaining 3D point cloud in the 2D horizontal plane of the reference map (see Figure 11), and (7) apply the ICP algorithm for self-location with this projected scan.

**Figure 12:**
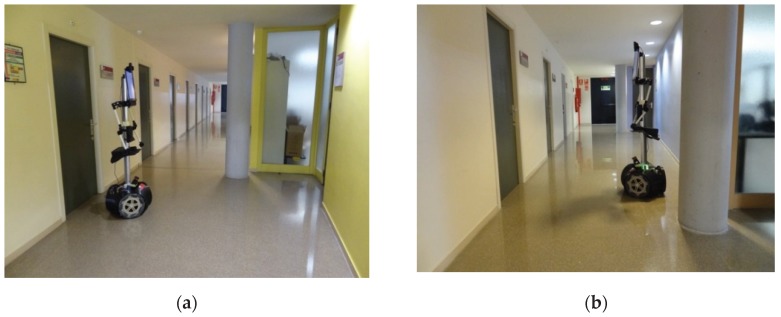
Mobile robot APR-03 in the corridor: (**a**) at the right side and (**b**) at the left side.

**Figure 13:**
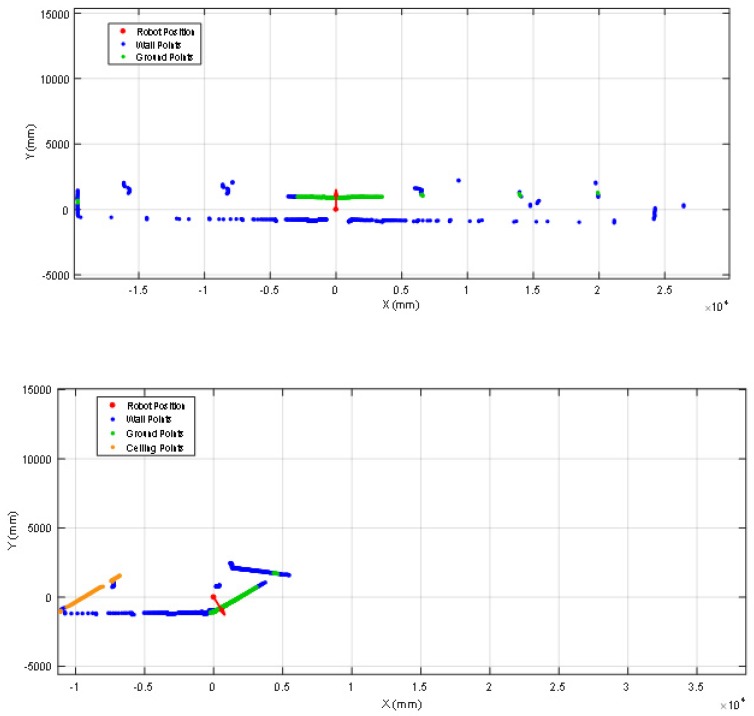
2D projection of some scans gathered by the LIDAR placed at the starting positions shown in Figure 12: (top) right side of the corridor and (down) left side of the corridor.

**Figure 14:**
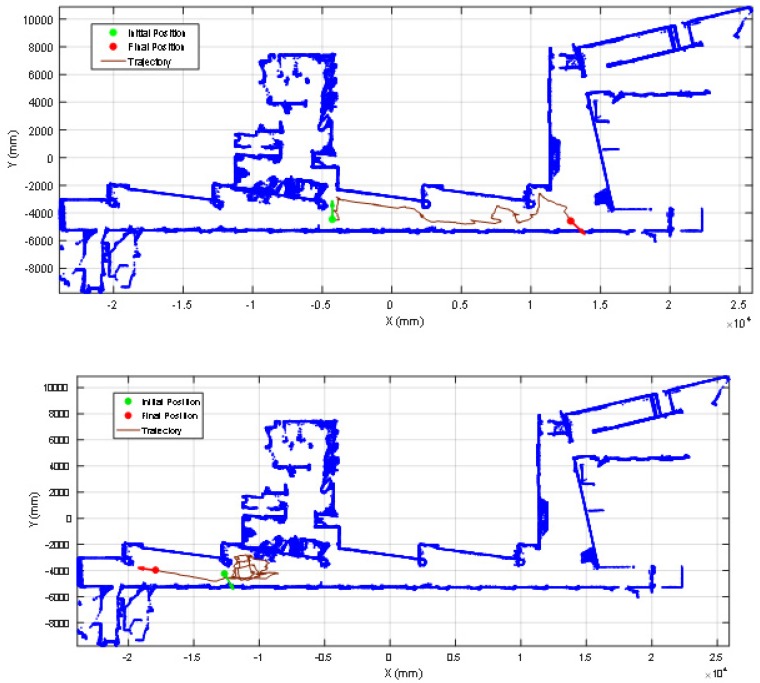
Estimated trajectory of the mobile robot with the 2D LIDAR tilted down 25° for the cases shown in Figure 12: (top) right side of the corridor and (down) left side of the corridor.

Figure 12 show the starting positions of two representative validation experiments. In the experiment shown in Figure 12a the mobile robot was placed and started in one side of the main corridor of the facility, approximately in the middle of the corridor. Figure 13 (top) show the projection of the first scan data gathered by the LIDAR. This figure includes the ground points (in green color) to evidence the effect of tilting the LIDAR. Figure 13 (top) shows that the LIDAR provides long distance information covering one side of the corridor, both ending areas of the corridor and also the location of many columns in between. Therefore, there are a large number of features that will reduce the uncertainty in the application of the ICP algorithm for self-location in the 2D reference map. Figure 14 (top) summarizes the estimated trajectory followed by the mobile robot APR-03 during this first experiment. The visual analysis of the real and estimated mobile robot trajectory can conclude that even though there were a few situations where the robot miscalculated its position, the ICP algorithm by itself had the capacity to recover and estimate a representative trajectory of the path followed by the mobile robot.

Alternatively, in the experiment shown in Figure 12b the mobile robot started on the opposite side of the corridor. Unexpectedly, in this starting position the LIDAR provides only short distance information, challenging the self-location due to the lack of relevant features gathered. Figure 13 (down) shows an example of scan data gathered by the LIDAR in this experiment. This figure depicts the ground points (in green color) and ceiling pints (in brown color). Figure 13 (down) shows that the LIDAR detects only a small part of the corridor without detecting the ends of the wall. This orientation scenario causes the sensor to gather less information, behaving like a 2D LIDAR with a lower distance range. Therefore, in this case the ICP algorithm has few features to match and the same features (one column, one plain wall and one inclined wall) are duplicated in different parts of the corridor of the facility, adding uncertainty to the self-location that may require the application of additional robust fusion approaches [32]. However, in the practice, a small displacement or turn of the mobile robot usually provides additional contour features and the uncertainty of the self-location procedure is significantly reduced. Figure 14 (bottom) shows the estimated trajectory followed by the mobile robot APR-03 in this second experiment. The visual analysis of the real and estimated mobile robot trajectory can conclude that the estimated trajectory is representative of the real mobile robot trajectory. Similar conclusions have been obtained in all additional experiments performed with starting points located in random points of the corridor of the facility.

**Figure 15:**
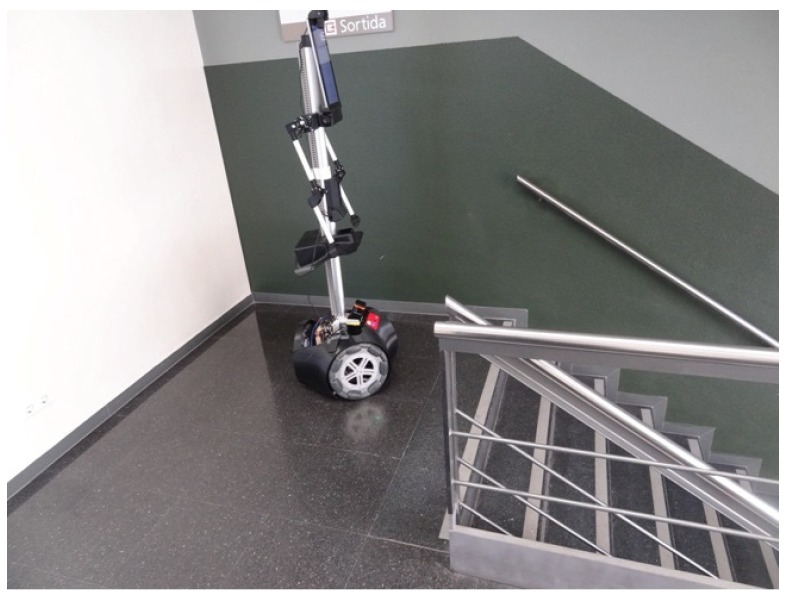
Image of the mobile robot APR-03 at the stair area of the facility.

**Figure 16:**
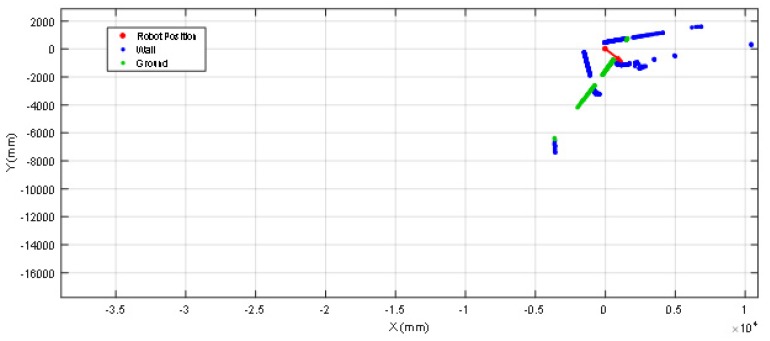
2D projection of a scan gathered by the LIDAR placed at the starting position shown in Figure 15. The arrow depicts the location and orientation of the mobile robot, the green dots depict scan points classified as ground points.

**Figure 17:**
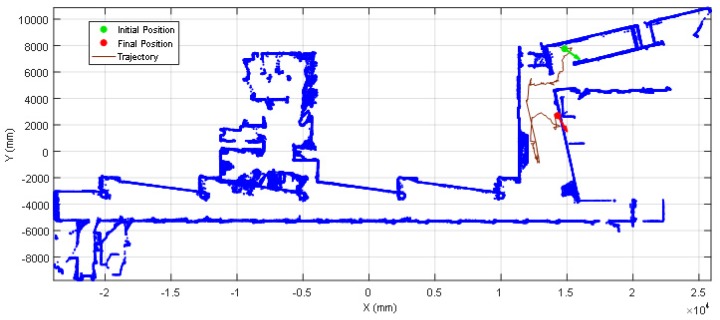
Estimated trajectory of the mobile robot with the 2D LIDAR tilted down 25° in one experiment performed in the stairs area of the facility.

Finally, Figure 15 shows the starting point of another validation experiment developed at the stair area of the facility, placing the mobile robot just in front of a stair going down. This area of the facility is very challenging for self-location because of the stairs going down and up and also because one wall is made of transparent glass that remains partially undetected by the LIDAR. Figure 16 shows the 2D projected scan data gathered by the LIDAR, this figure still includes the ground points (in green color) to evidence the effect of tilting the LIDAR. Figure 16 shows that the LIDAR detects ground points only on its left-front so the collision avoidance algorithm (which operates isolated from the ICP algorithm) can detect and avoid the hole of the stair even in case of an incorrect self-location in this area of the facility. Even in this case, the ICP algorithm has detected enough features in order to self-locate successfully the mobile robot in the 2D reference map. Finally, Figure 17 shows the estimated pseudo-random trajectory followed by the mobile robot APR-03 in this experiment. The visual analysis of the real and estimated mobile robot trajectory can conclude that the estimated trajectory is representative of the real mobile robot trajectory. Similar conclusions have been obtained in all additional experiments performed with starting points located in random points of the stair area of the facility.

## 5. Discussion

The proposal of this paper has been inspired on the work of Takahashi et al. [16] who proposed the development of an emergency obstacle avoidance procedure for a mobile robot using a fixed tilted down 2D LIDAR sensor. The experiments performed in Section 3 of this paper have validated this proposal and has also presented how some common objects and obstacles are scanned by a 2D push-broom LIDAR. The objects used in the validation experiments have been a small box placed on the ground, one shoe/leg, stair going down, and stair going up.

The use of a 2D LIDAR tilted-down provides information of the ground plane in front of the mobile robot and enables the detection of objects or holes by the application of a threshold to the relative ground height. In this paper a positive threshold of 50 mm over the expected ground height has been used to detect obstacles and a negative threshold of −50 mm to detect holes. In some tilt cases (Table 3, less than 15°) the reflection of the laser beam in the ground can be identified as a hole in front of the mobile robot instead of an object.

The results of Figure 4 show that a shoe appears in the scan points like a small object while the leg appears like a big object that is always detected by a 2D LIDAR regardless of the tilt. The complete detection results of Table 3 also show that small incidence angles produce reflections and incorrect distance measurements. The results of Figure 5 show that the ground is not detected when having a going down stair in front of the mobile robot. The complete detection results of Table 4 can be used to estimate the minimum tilt angle of the 2D LIDAR in order to stop a mobile robot before falling down the stairs. The results of Figure 6 and Table 5 show that a going up stair is always detected by a 2D LIDAR but the accuracy of the detection increases as the tilt angle increases.

According these detection results, the optimal fixed tilt angle for a 2D LIDAR model UTM-30LX mounted in the mobile robot APR-03 is 25°. This conclusion has been tested with a set of experiments in which the mobile robot APR-03 moved pseudo-randomly through an area of the experimentation facility where there were stairs. In all these experiments the mobile robot successfully detected the stairs going down and up, navigating without any collision or noticeable problem while maintaining a safe distance with all obstacles and furniture elements. The optimal push-broom angle depends on the height of the measurement plane and the velocity of the mobile robot. In any case, a fixed 2D push-broom LIDAR must supervise the ground in front of the mobile robot, avoid collisions with objects placed on the ground and avoid falling downstairs.

In a complementary way, the experiments described in Section 5 of this paper have confirmed that the information gathered by a 2D push-broom LIDAR can be used to self-locate a mobile robot using a 2D map obtained previously with the same 2D LIDAR placed in horizontal. The influence of the distance range of the 2D LIDAR in the self-location results has not been evaluated although worse location results must be expected when using a 2D LIDAR with a lower distance range. In the cases analyzed the mobile robot was started in random locations of the corridor of the facility. The worst self-location results have been obtained in specific locations in which the tilted 2D LIDAR provides only short-range information of the scenario.

Finally, Figure 15, Figure 16 and Figure 17 describe one of the experiments in which the mobile robot APR-03 performed a pseudo-random displacement in an area of the facility with stairs and a large lateral transparent wall. All of these obstacles were located in a relatively small area. Such conditions are a real challenge for any mobile robot because there is a real risk of falling down the stairs and to collide with the stairs going up or the transparent wall. On the one hand, the mobile robot APR-03 has avoided any collision or falling thanks to the information gathered with the tilted 2D LIDAR. On the other hand, the information gathered with the tilted 2D LIDAR has been processed and used to self-locate the mobile robot using the ICP algorithm and the information provided by a 2D horizontal map. This map was created previously in another exploration experiment with a mobile robot mounting a 2D horizontal LIDAR.

## 6. Conclusions

The main conclusion of this paper is that an onboard 2D push-broom LIDAR with a tilt angle of 25° provides enough information for collision avoidance and for mobile robot self-location. The advantage of this proposal is the avoidance of using a motorized device to change the tilt of the LIDAR in order to combine these two characteristics in a mobile robot. In this paper, the self-location procedure was implemented using a reference 2D map obtained previously using the same 2D LIDAR placed horizontally (with a tilt angle 0°). The successful validation of the hypothesis of this paper has been possible because (1) the large distance range of the 2D LIDAR model used and (2) because the experimentation facility has large walls, plain doors and some big fixed furniture objects that are not prone to environmental changes.

The use of a 2D push-broom LIDAR has the drawback of reducing the information gathered from the front of the mobile robot. In the case of the APR-03 using the UTM-30LX the frontal detection range was reduced from 30,000 mm (available with a tilt angle of 0°) to 750 mm (available with a tilt angle of 25°). The availability of a large distance range is ideal to create accurate maps using SLAM but a LIDAR with a tilt angle of 0° does not provide information of small objects placed in the ground and does not detect the hole and the danger that represents a stair going down.

Future work will be focused on applying the 2D push-broom LIDAR in domestic scenarios and recognize user activity [33] in a smart home.

## Figures and Tables

**Table 1:** Illustrative images of the validation detection experiments: small box, one shoe/leg, stair going down, and stairs going up.

Obstacle	Illustrative Image
Small box on the floor	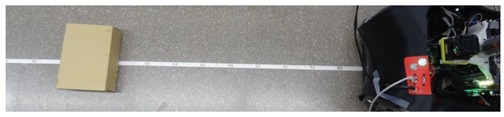
One shoe/leg	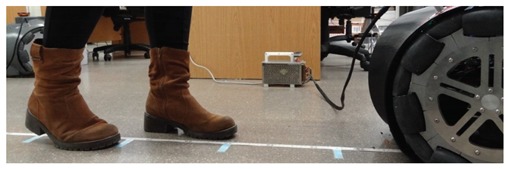
Stairs going down	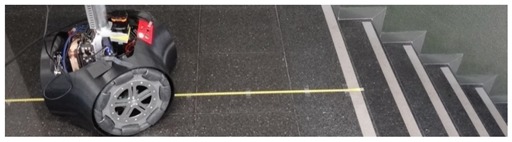
Stairs going up	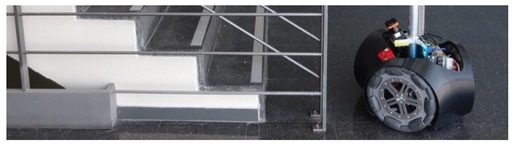

**Table 2:** Small box detection results obtained for different distances and LIDAR tilt angles: (√) object detected, (R) object detected after ground reflection, (-) object not detected.

	Tilt									
Distance	0°	5°	10°	15°	20°	25°	30°	35°	40°	45°
250 mm	-	-	-	-	-	-	-	-	√	√
500 mm	-	-	-	-	-	√	√	√	-	-
750 mm	-	-	-	-	√	√	-	-	-	-
1000 mm	-	-	-	√	√	-	-	-	-	-
1250 mm	-	-	-	√	-	-	-	-	-	-
1500 mm	-	-	√	√	-	-	-	-	-	-
1750 mm	-	-	√	-	-	-	-	-	-	-
2000 mm	-	-	√	-	-	-	-	-	-	-
2250 mm	-	-	R	-	-	-	-	-	-	-
2500 mm	-	-	R	-	-	-	-	-	-	-
2750 mm	-	-	-	-	-	-	-	-	-	-
3000 mm	-	√	-	-	-	-	-	-	-	-
3250 mm	-	√	-	-	-	-	-	-	-	-
3500 mm	-	√	-	-	-	-	-	-	-	-
3750 mm	-	√	-	-	-	-	-	-	-	-
4000 mm	-	√	-	-	-	-	-	-	-	-

**Table 3:** One shoe/leg detection results obtained for different distance and LIDAR tilt angles: (√) object detected, (R) object detected after ground reflection, (-) object not detected.

	Tilt									
Distance	0°	5°	10°	15°	20°	25°	30°	35°	40°	45°
250 mm	√	√	√	√	√	√	√	√	√	√
500 mm	√	√	√	√	√	√	√	√	-	-
750 mm	√	√	√	√	√	√	-	-	-	-
1000 mm	√	√	√	√	√	-	-	-	-	-
1250 mm	√	√	√	√	-	-	-	-	-	-
1500 mm	√	√	√	-	-	-	-	-	-	-
1750 mm	√	√	√	-	-	-	-	-	-	-
2000 mm	√	√	√	-	-	-	-	-	-	-
2250 mm	√	√	R	-	-	-	-	-	-	-
2500 mm	√	√	R	-	-	-	-	-	-	-
2750 mm	√	√	R	-	-	-	-	-	-	-
3000 mm	√	√	R	-	-	-	-	-	-	-
3250 mm	√	√	R	-	-	-	-	-	-	-
3500 mm	√	√	R	-	-	-	-	-	-	-
3750 mm	√	√	-	-	-	-	-	-	-	-
4000 mm	√	√	-	-	-	-	-	-	-	-

**Table 4:** Stairs going down detection results obtained for different distances and LIDAR tilt angles: (S) hole or stair going down detected, (G) ground detected at the expected location, (numeric value) average distance of a frontal obstacle.

Down Stairs Distance	Tilt									
	0°	5°	10°	15°	20°	25°	30°	35°	40°	45°
250 mm	5632 mm	S	S	S	S	S	S	S	S	S
500 mm	5856 mm	S	S	S	S	S	S	S	G	G
750 mm	6071 mm	S	S	S	S	S	G	G	G	G
1000 mm	6340 mm	S	S	S	S	G	G	G	G	G
1250 mm	6642 mm	S	S	S	G	G	G	G	G	G
1500 mm	6907 mm	S	S	G	G	G	G	G	G	G

**Table 5:** Stairs going up detection results obtained for different distances and LIDAR tilt angles: (G) ground detected at its expected location, (numeric value) average distance in mm of the frontal obstacle detected.

Up Stairs Distance	Tilt									
	0°	5°	10°	15°	20°	25°	30°	35°	40°	45°
250 mm	780	492	510	499	515	430	350	270	265	251
500 mm	1012	729	764	759	575	500	479	500	G	G
750 mm	1305	1036	1010	787	739	765	G	G	G	G
1000 mm	1596	1291	1254	984	1001	G	G	G	G	G
1250 mm	1812	1513	1267	1239	G	G	G	G	G	G
1500 mm	2042	1746	1151	G	G	G	G	G	G	G

## Supplementary Materials

The dataset containing the 2D LIDAR scans used in this paper is available online at https://www.mdpi.com/1424-8220/20/9/2500/s1. This zip file has approximately 30 MB and includes a menu-driven MATLAB file (main.m) for easy data selection and visualization.

## Abbreviations

APR	Assistant Personal Robot
ICP	Iterative Closest Point
LIDAR	Ligth Detection and Ranging
SLAM	Simultaneous Localization and Mapping
